# Kidney involvement of chronic active Epstein‐Barr virus infection

**DOI:** 10.1002/jha2.641

**Published:** 2023-01-20

**Authors:** Ami Fukumoto, Shinnosuke Sugihara, Kurumi Seki, Masami Takeuchi, Tomo Suzuki, Matsue Kosei

**Affiliations:** ^1^ Division of Hematology/Oncology Department of Internal Medicine Kameda Medical Center Kamogawa Japan; ^2^ Division of Nephrology Department of Internal Medicine Kameda Medical Center Kamogawa Japan; ^3^ Department of Clinical Pathology Kameda Medical Center Kamogawa Japan

**Keywords:** chronic active epstein‐barr virus infection, tubulointerstitial nephritis

1

A 54‐year‐old Japanese woman presented with a fever of unknown origin and renal dysfunction. She had experienced intermittent fever for 10 years, but the frequency increased to a constant fever. Her renal function had gradually deteriorated over the past year, and serum creatinine and urinary α1‐microglobulin levels were elevated to 2.23 mg/dL and 113 mg/L, respectively. Physical and laboratory examinations were unremarkable, except for mild anemia and elevated serum creatinine levels. Analysis of peripheral blood lymphocyte subsets showed 59% CD3, 35% CD4 and 31% CD8. Epstein‐Barr virus (EBV) serology showed elevated levels of EBV capsid antigen IgG and EBV nuclear antigen (1:1280 and 1:40, respectively). Plasma EBV‐DNA level was 5.63 log IU/mL, and an oligoclonal band was detected by Southern blot hybridization of EBV terminal repeat analysis. Quantification of EBV‐DNA in peripheral lymphocyte subset using magnetic beads showed that EBV predominantly resided in CD4+ T‐cells. (CD4, 258,768; CD8, 27,738; and CD56, 23,369; IU/µgDNA, respectively). T‐cell receptor α gene rearrangement was undetected. Based on the above, she was diagnosed with chronic active EBV infection (CAEBV). ^18^F‐fluorodeoxyglucose positron emission tomography/computerized tomography showed bilateral kidney enlargement and mildly enhanced uptake (maximum standardized uptake value 4.08 g/mL) (Figure 1 top left). Kidney biopsy specimen stained with hematoxylin and eosin showed diffuse tubulointerstitial infiltration of small to medium‐sized lymphocytes in the renal parenchyma (Figure 1 top right). Massive infiltration of CD4+ T‐cells was documented by immunohistochemical staining (Figure 1 bottom left). Furthermore, EBV‐infected cells were detected by in situ hybridization with EBV‐encoded RNA (EBER) (Figure 1 bottom right). Immunostaining of EBV nuclear antigen‐2 and latent membrane protein 1 were negative. EBER‐positive cells were distributed in the same region as CD4+ T‐cells, consistent with the predominant presence of EBV‐DNA in CD4+ T‐cells. Her renal function had improved after initiating chemotherapy, and she is scheduled for hematopoietic stem cell transplantation.

**FIGURE 1:**
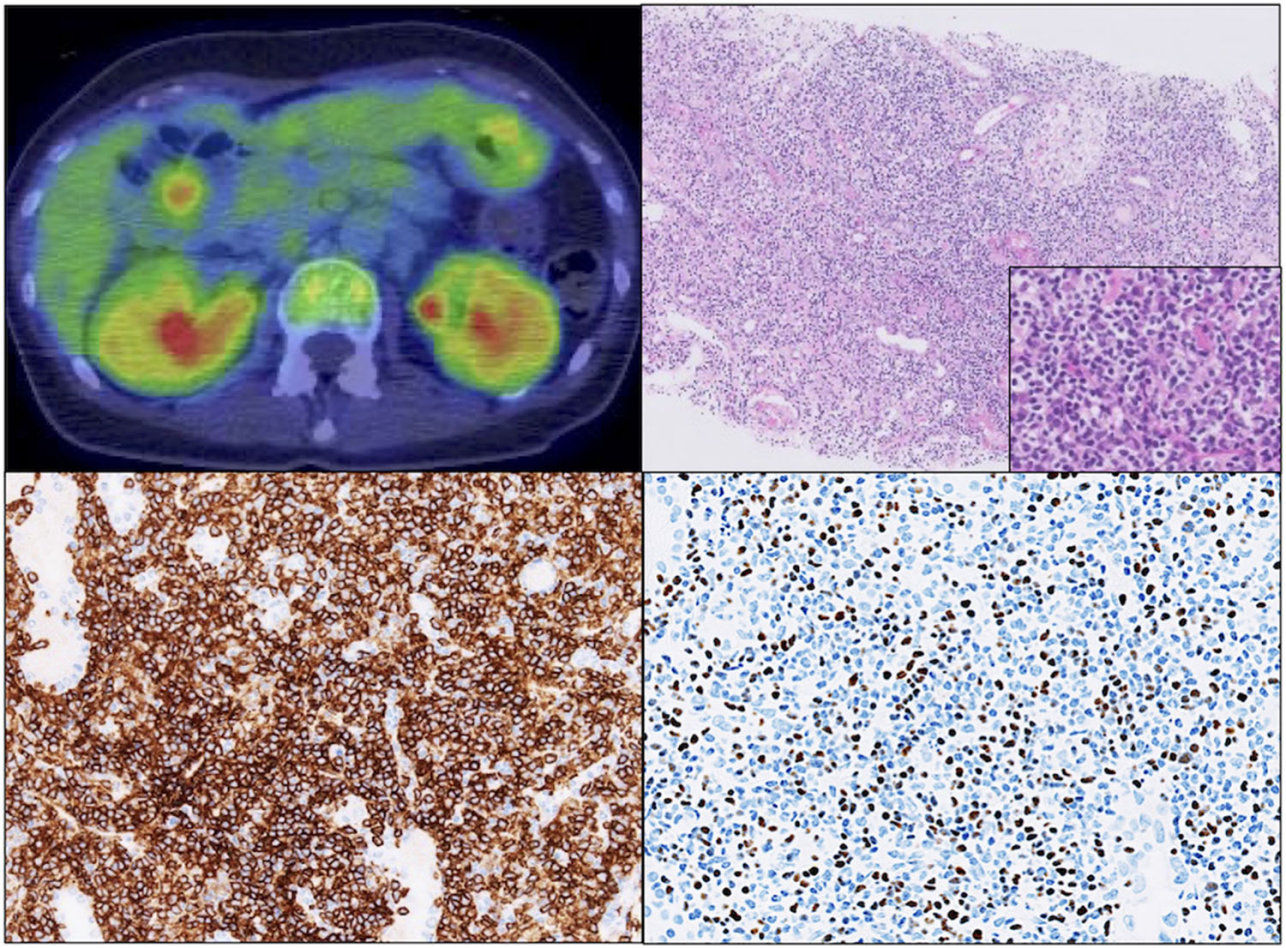
Top left; 18F‐fluorodeoxyglucose positron emission tomography/computerized tomography (FDG‐PET/CT) shows mildly enhanced uptake in the bilateral kidneys (maximum standardized uptake value 4.08 g/mL). Top right; kidney biopsy specimen with hematoxylin and eosin staining shows diffuse infiltration of small to medium‐sized lymphocytes. Bottom left; immunohistochemical staining shows massive infiltration of CD4+ T‐cells. Bottom right; diffuse infiltration of Epstein‐Barr virus (EBV)‐positive cells is noted in the tubulointerstitial space

The clinical features of EBV infection vary according to the type of EBV‐infected cells. The predominant EBV‐infected cells in EBV‐induced hemophagocytic lymphohistiocytosis are CD8+ T cells, whereas in CAEBV, CD4+ T cells are predominantly infected. Although rare, renal invasion of EBV should be considered in cases with renal impairment, and its clinical significance needs to be investigated.

## AUTHOR CONTRIBUTIONS

AF and SS wrote and edited the manuscript, provided patient care and reviewed the literature. KS and MT provided patient care. TS and KM wrote and edited the manuscript. All authors reviewed and approved the final version of the manuscript

## CONFLICT OF INTEREST

The authors declare they have no conflicts of interest.

## FUNDING INFORMATION

The authors received no specific funding for this work.

## ETHICS STATEMENT

This study was conducted according to the Declaration of Helsinki, and written consent was obtained from the patient for this report. The patient provided written informed consent for the publication of this case, with the removal of all identifying information to ensure anonymity and maintain privacy.

## Supporting information




Supporting information

## Data Availability

The data that support the findings of this study are available from the corresponding author upon reasonable request.

